# Facile synthesis of hierarchical CNF/SnO_2_/Ni nanostructures via self-assembly process as anode materials for lithium ion batteries

**DOI:** 10.1098/rsos.171522

**Published:** 2018-06-20

**Authors:** Haitong Tang, Xinru Yu, Shi Jin, Fanling Meng, Yan Yan, Zhongmin gao

**Affiliations:** 1 Key Laboratory of Automobile Materials, Ministry of Education, College of Materials Science and Engineering, Jilin University, Changchun, 130012, People's Republic of China; 2 State Key Laboratory of Inorganic Synthesis and Preparative Chemistry, College of Chemistry, Jilin University, Changchun, 130012, People's Republic of China; 3 Jilin Jianzhu University, Changchun, 130018, People's Republic of China

**Keywords:** lithium ion battery, SnO_2_, polyacrylonitrile, carbon nanofibre, hierarchical, self-assembly

## Abstract

Hierarchical carbon nanofibre (CNF)/SnO_2_/Ni nanostructures of graphitized carbon nanofibres and SnO_2_ nanocrystallines and Ni nanocrystallines have been prepared via divalent tin–alginate assembly on polyacrylonitrile (PAN) fibres, controlled pyrolysis and ball milling. Fabrication is implemented in three steps: (1) formation of a tin–alginate layer on PAN fibres by coating sodium alginate on PAN in a water medium followed by polycondensation in SnCl_2_ solution; (2) heat treatment at 450°C in a nitrogen atmosphere; (3) ball milling the mixture of CNF/SnO_2_ fibres and Ni powder. The CNF/SnO_2_/Ni nanocomposite exhibits good lithium ion storage capacity and cyclability, providing a facile and low-cost approach for the large-scale preparation of anode materials for lithium ion batteries.

## 1. Introduction

Research into rechargeable lithium ion batteries (LIBs) has played a vital role in sustainable energy storage devices to meet the higher expectation of electric vehicles and portable products [1–6]. For commercial LIBs, graphite with a theoretical capacity of 372 mAh g^−1^ is employed as the anode material, which is not able to satisfy the energy demand for the new technology [7–8]. In addition, even though alloy and dealloy materials have high capacity and high energy density, electrode pulverization and separation from the larger volume effect during discharge and charge lead to poor cycling [9]. Therefore, there is a requirement to develop large-scale commercial anode materials with high capacity and low fabrication cost. Recently, tin oxides (SnO_2_) and tin sulfides (SnS*_x_ x* = 1–2) with different structure have attracted significant attention due to their high energy density and low cost [10–21]. However, poor cycling and mass production hamper their practical applications to some extent. Recent efforts to address the above-mentioned issues have led to the development of SnO_2_ nanobox, nanotube, nanosheet, hollow nanostructure and an SnO_2_/graphene hybrid nanocomposite [22–31]. Although the carbon nanofibre/SnO_2_ (CNF/SnO_2_) nanocomposites show specially improved performance as LIB anode materials, they were generally fabricated via small-scale production with high cost [14,32,33]. Furthermore, Ni nanocrystals can act as a buffer to relieve the mechanical stress induced by the large volume changes upon cycling, and can also help in the successful suppression of Sn coarsening during cycling and consequent enhancement of the stability of the reversible conversion reactions [34,35]. Thus, it is highly desirable to prepare the carbon nanofibre/tin oxide/nickel (CNF/SnO_2_/Ni) nanocomposite with hierarchical nanostructure and long-term cycling stability via facile and scalable methods.

Here, we report a facile and low-cost approach for the preparation of hierarchically graphitized carbon nanofibres and SnO_2_ nanocrystals and Ni nanocrystals by using commercial polyacrylonitrile (PAN) fibres as the precursor. The process of fabrication is done in three steps: (1) controlled formation of a tin–alginate layer on PAN by dispersing PAN fibres into a solution of sodium alginate, forming PAN/sodium alginate composites, then converting to PAN/tin–alginate nanocomposites by dispersing them in SnCl_2_ solution; (2) controlled pyrolysis of the hybrid nanocomposites at 450°C in a nitrogen atmosphere, giving rise to the nanocomposite of graphitized carbon and crystalline SnO_2_; (3) ball milling the mixture of CNF/SnO_2_ nanofibres and Ni powder to fabricate the hierarchical CNF/SnO_2_/Ni nanostructure. The CNF/SnO_2_/Ni nanocomposite exhibits good lithium ion storage capacity and stable cyclability as the anode materials for LIBs. This work provides a facile and low cost as well as large-scale production approach for preparation of anode materials for LIBs.

## 2. Material and methods

### 2.1. Materials

Commercial PAN fibres were purchased from Jilin chemical fibre group Co., Ltd. Tin tetrachloride (SnCl_4_) was purchased from Sinopharm chemical reagent Co., Ltd. Tin powder (Sn) was purchased from Shanghai Aladdin Biochemical Technology Co., Ltd. Sodium alginate was purchased from Sigma-Aldrich. All the chemical reagents used were of analytical grade without any purification process.

### 2.2. Preparation of the hierarchical CNF/SnO_2_/Ni nanocomposite

In a typical experiment, 0.5 g PAN fibres were cut into small pieces with a length of approximately 0.5 cm. Monodispersed PAN should be dispersed in water. Then, the mixtures were heated at a temperature ranging from 70°C to 90°C. This process is very important, as it can effectively activate the surficial groups to link the sodium alginate. Subsequently, The PAN nanofibres were mixed with sodium alginate solution (1 mol l^−1^, 200 ml) and stirred for 3 h at 90°C to attach sodium alginate on the surface of PAN. Next, the product was washed using absolute ethyl alcohol and then submerged in a solution of SnCl_2_ (0.04 mol l^−1^, 200 ml), obtained from SnCl_4_ and Sn powder by dispersing 2.37 g Sn powder into SnCl_4_ solution (0.02 mol l^−1^, 200 ml) and stirring for 12 h, for 5 h to obtain the PAN/tin–alginate. The process was repeated four times to enhance the thickness of the tin–alginate shell. Next, the as-synthesized products were calcined at 450°C for 2 h in a muffle furnace at a heating rate of 1°C min^−1^ in a nitrogen atmosphere to form the carbon nanofibre/SnO_2_ composites (CNF/SnO_2_). The CNF/SnO_2_/Ni nanostructure was obtained by ball milling the mixture of CNF/SnO_2_ and nickel (Ni) powder with different weight ratio in air for 15 h at room temperature.

### 2.3. Characterization

The XRD patterns of the CNF/SnO_2_/Ni nanocomposites were recorded on a Rigaku D/max 2550 X-ray diffractometer, using a monochromatized Cu target radiation resource (*λ* = 1.54 Å). The morphology of CNF/SnO_2_/Ni nanocomposite was analysed by using a JEOL-6700F field-emission scanning electron microscope (FESEM) at an accelerating voltage of 3 kV. The element mappings of Sn, O, C and Ni were conducted by using a JEOL-7800F FESEM with an energy dispersive X-ray spectroscopy (EDS) attachment at an accelerating voltage of 20 kV. The transmission electron microscopy (TEM) images were taken on a FEI Tecnai G2S-Twin with an EDS attachment and field-emission gun operating at 200 kV. The X-ray photoelectron spectra (XPS) were recorded on an ESCAlab 250 Analytical XPS spectrometer. Thermogravimetric analysis (TGA) was carried out in air in the temperature range 30–850°C using a Q500 thermogravimetric analyser instrument. Raman spectroscopy was performed in a Renishaw inVia microRaman system with an internal 532 nm laser source. The nitrogen adsorption–desorption isotherms were recorded at 77.3 K with a Micromeritics ASAP 2420 surface area and porosity analyser. The batteries were assembled in an argon-filled glove box using lithium metal as counter electrode, where oxygen and moisture levels were controlled to less than 0.1 ppm, and the electrolyte was 1 M LiPF_6_ in a mixture of ethylene carbonate : dimethyl carbonate (1 : 1 by v/v). The CNF/SnO_2_/Ni nanocomposite was used as working electrodes. The electrochemical performance was conducted with galvanostatic charge and discharge on a LAND CT2001A cell test apparatus in the voltage range 0.1–3.0 V at room temperature. Cyclic voltammetry was performed on a VSP multichannel potentiostat–galvanostat at a scan rate of 0.1 mVs^−1^. Electrochemical impedance spectroscopy (EIS) was also performed with the VSP instrument.

## 3. Results and discussion

**Figure 1.**
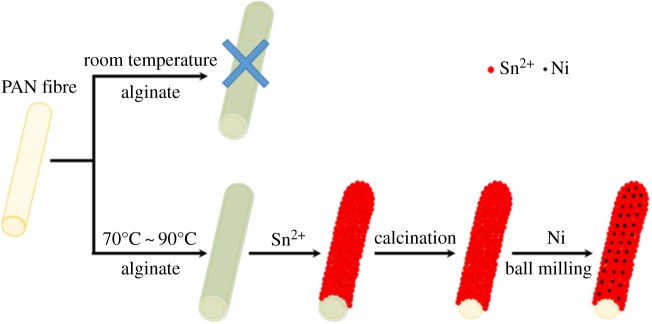
The process of synthesis of hierarchical CNF/SnO_2_/Ni nanocomposite.

The fabrication process of the CNF/SnO_2_/Ni nanocomposite is illustrated in figure 1. A series of experiments have been conducted to optimize the structure, morphology and electrical properties. It can be found that monodispersed PAN/sodium alginate can be obtained via dispersing monodispersed PAN in water and heating the mixture at a temperature ranging from 70 to 90°C. However, it cannot be obtained at room temperature, which indicates that the heating process can effectively activate the surficial group to link sodium alginate. Then, PAN/tin–alginate nanocomposite can form via an ion exchange process through dispersing PAN/sodium alginate into a solution of SnCl_2_ with stirring for 3 h at room temperature as the divalent metal ion can be abundantly immobilized in alginate. Next, the as-synthesized products were calcined at 450°C for 2 h in a muffle furnace at a heating rate of 1°C min^−1^ in a nitrogen atmosphere to form the carbon nanofibre/SnO_2_ composites (CNF/SnO_2_). Finally, the CNF/SnO_2_/Ni nanostructures can be obtained by ball milling the mixture of CNF/SnO_2_ and Ni powder with different weight ratios in an air atmosphere for 15 h at room temperature. Finally, the Ni nanoparticles can be assembled into the CNF/SnO_2_ nanostructure by means of ball milling treatment.

**Figure 2.**
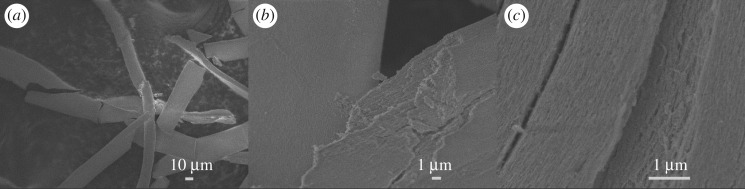
FESEM images of (*a*) fibre-like CNF/SnO_2_ nanocomposite, (*b*) amplified CNF/SnO_2_ with layer structure and (*c*) surficial groove structure of CNF/SnO_2_ nanocomposite.

**Figure 3.**
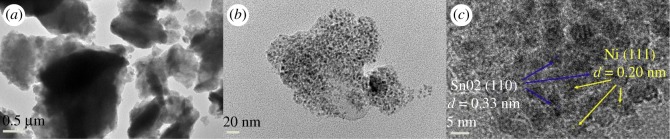
TEM images of (*a*) CNF/SnO_2_/Ni hierarchical nanocomposite, (*b*) amplified CNF/SnO_2_/Ni hierarchical nanostructure and (*c*) HRTEM image of CNF/SnO_2_/Ni hierarchical nanostructure.

FESEM images show that CNF/SnO_2_ nanostructures hold fibre-like morphology and the surface of CNF is covered with several layers of SnO_2_ (figure 2*a,b*). The CNF/SnO_2_ nanocomposite is fragile and can be ground into powder (electronic supplementary material, figure S1a). The CNF/SnO_2_ nanostructure consists of a CNF core and SnO_2_ shell with groove structure (figure 2*c*), which should be attributed to the original texture of PAN (electronic supplementary material, figure S1b). It indicates that the groove structure and nanopores on the surface of PAN can offer the sites to connect the SnO_2_ and Ni nanoparticles. Comparing with CNF/SnO_2_ nanostructure, the CNF/SnO_2_/Ni nanocomposite is a pulverized powder (figure 3*a*), because it is obtained through the ball milling process. TEM analysis suggests that Ni nanoparticles can be assembled into the CNF/SnO_2_ nanostructure (figure 3*b*) and ball milling treatment cannot break the groove structure, which causes the CNF/SnO_2_/Ni nanocomposite to be porous. A HRTEM image taken from the edge of CNF/SnO_2_/Ni nanocomposite reveals that the d-spacing between two consecutive planes are about 0.33 and 0.20 nm, respectively, according to the d_110_ and d_111_ values of SnO_2_ (tetragonal) and Ni (cubic) phase, respectively (figure 3*c*).

**Figure 4.**
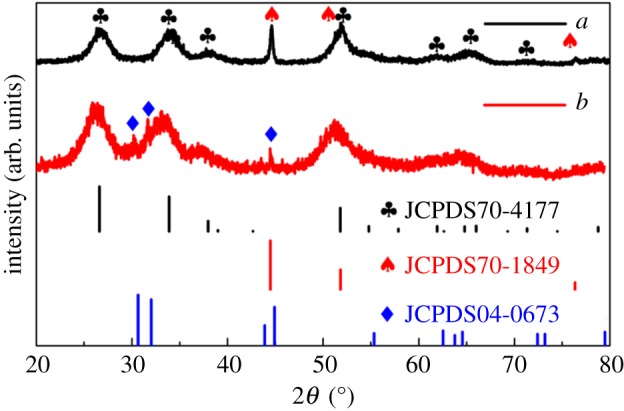
XRD pattern of the as-synthesized samples: (*a*) CNF/SnO_2_/Ni hierarchical nanocomposite and (*b*) CNF/SnO_2_ nanocomposite.

The crystal structures of the CNF/SnO_2_ and CNF/SnO_2_/Ni nanocomposites were characterized by XRD (figure 4), which can be indexed to the polycrystalline tetragonal SnO_2_ (JCPDS no. 70-4177) and cubic Ni (JCPDS no. 70-1849), respectively. In addition, for CNF/SnO_2_, even though the major peaks can be indexed to reflections of tetragonal SnO_2_, the peak at 30.64° is indexed to (200) reflection of metallic Sn (tetragonal), confirming that a small quantity of divalent tin ions are reduced into metallic Sn. It is interesting to note that reflection of metallic Sn disappears after the ball milling process, which indicates that metallic Sn is oxidized into SnO_2_ via the ball milling process. This result shows that Ni nanoparticles can be assembled into CNF/SnO_2_ to generate CNF/SnO_2_/Ni nanocomposites via the ball milling process. In addition, the EDS mapping of CNF/SnO_2_/Ni samples have been performed to investigate the distribution of C, Sn, Ni and O element (electronic supplementary material, figure S2a,b). These results show that the elements are uniformly distributed in the samples.

TGA data of CNF/SnO_2_/Ni result shows a major weight loss of 30 wt% ranging from 200 to 800°C (electronic supplementary material, figure S3a), which can be attributed to the removal of the partial carbon because the CNF from pyrolysed PAN cannot be removed completely at 800°C. The structure of the CNF/SnO_2_/Ni was further investigated by Raman spectroscopy (electronic supplementary material, figure S3b). Two broad peaks, located at around 1350 cm^−1^ (D band) and 1600 cm^−1^ (G band) are observed, corresponding to the vibration of amorphous and graphitized carbon, respectively. The intensity ratio of D to G (*I*_D_/*I*_G_) is determined to be 1.46 for CNF/SnO_2_/Ni, suggesting that the carbonization of PAN fibres and formation of SnO_2_ may take place concurrently.

**Figure 5.**
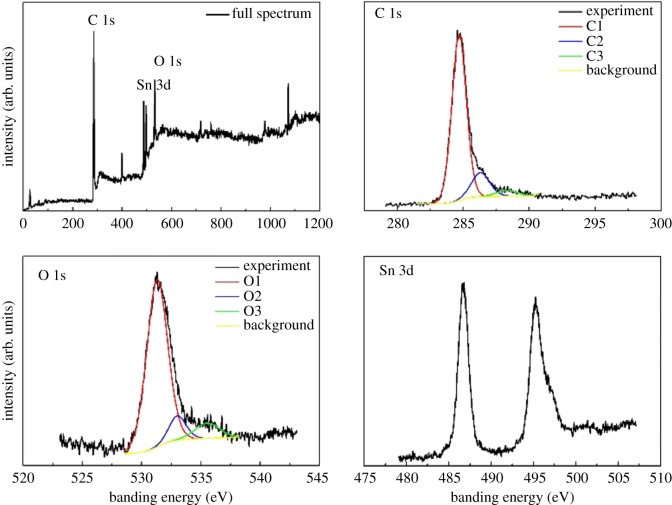
XPS spectra of the CNF/SnO_2_/Ni hierarchical nanocomposite. (*a*) Survey spectrum, (*b*) C 1s, (*c*) O 1s and (*d*) Sn 3d.

XPS was applied to study the surface composition, interfacial interactions and chemical nature of the CNF/SnO_2_ and CNF/SnO_2_/Ni nanocomposite, respectively. Figure 5*a* shows the survey spectrum of XPS analysis, confirming the presence of the elements C, N, O and Sn. The broad peak with an asymmetric tail toward higher binding energy of C1s implies plentiful sp^2^ carbon for CNF/SnO_2_/Ni, implying slight low content of sp^2^ carbon, in contrast to CNF/SnO_2_ (electronic supplementary material, figure S4). The deconvoluted XPS peaks of C 1s for CNF/SnO_2_/Ni nanocomposite display a unique quadruplet with binding energies at 284.7, 286.3 and 288.2 eV (figure 5*b*). They may be attributed to the graphitized carbon (C1) [36], adsorbed amorphous carbon (C2) [37] and the C–O bonds (C3) [38], respectively. The deconvoluted XPS peaks of O 1s are a triplet with binding energies of 531.3, 533.0 and 535.7 eV, which may be attributed to the lattice oxygen (O1) [39], the O–C bonds (O2) and the adsorbed oxygen (O3) [40], respectively (figure 5*c*). The deconvoluted XPS peaks of Sn 3d are a unique doublet at 486.9–495.6 eV (figure 5*d*), suggesting the presence of Sn^4+^ in CNF/SnO_2_/Ni nanocomposite and a slight shift to higher binding energy of both Sn 3d_5/2_ and Sn 3d_3/2_ [41], compared to those of CNF/SnO_2_. The shifts can be attributed to the oxidation state of Sn, conforming to the XRD characterization. These results show that SnO_2_ nanocrystals attach to carbon nanofibres strongly. The specific surface area and pore size distribution of the specific surface area and pore size distribution of the CNF/SnO_2_/Ni nanocomposite were calculated based on the nitrogen adsorption–desorption data (electronic supplementary material, figure S5). The nitrogen adsorption–desorption isotherm of CNF/SnO_2_/Ni exhibits a type III hysteresis loop, indicating the presence of hierarchical pores. The specific surface area is calculated to be 6.37 m^2^ g^−1^ by using the Brunauer–Emmett–Teller method. The pore size distribution, calculated using the Barrett–Joyner–Halenda model, suggests the presence of hierarchical porosity. The hierarchical pores might be formed by groove structures of SnO_2_ nanocrystallites.

**Figure 6.**
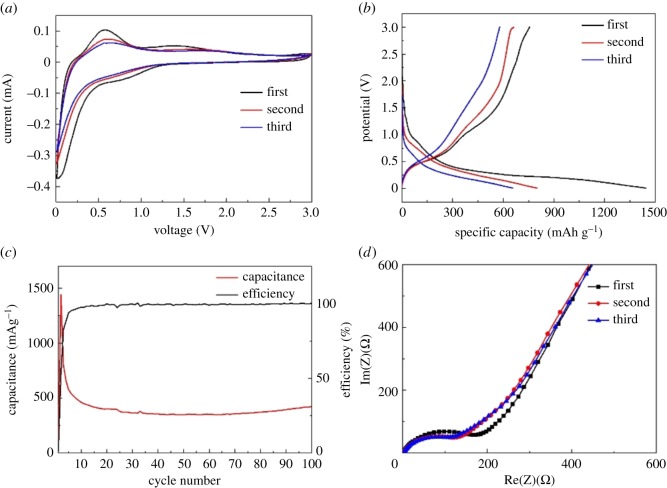
The performance of the CNF/SnO_2_/Ni hierarchical nanocomposite as anode materials for LIBs: (*a*) CV curves at a scan rate of 0.2 mV s^−1^ over a potential range of 0.01 to 3.0 V, (*b*) the charge–discharge cycle at a rate of 200 mAg^−1^ between 0.01 and 3.0 V, (*c*) cycling performance cycled at a rate of 200 mAg^−1^ and (*d*) impedance plot.

To evaluate electrochemical performance via galvanostatic discharge and charge process, the coin cells testing were fabricated by using CNF/SnO_2_/Ni as anode materials. Figure 6*a* exhibits the cyclic voltammograms (CVs) of the CNF/SnO_2_/Ni electrode at a scan rate of 0.2 mV s^−1^ over a potential range of 0.01 to 3.0 V for the first three cycles. The slope plateau ranging from 0.9 to 0.5 V can be attributed to reduction of SnO_2_, and partial slope plateau originates from the alloying between Sn and Li. The solid–electrolyte interphase (SEI) film on the electrode generates between 0.5 and 0.01 V in the first cathodic scan. Obviously, these processes are irreversible according to the following reaction:

3.1 $${\text{SnO}}_{2}+\text{Li}+\to \text{Sn}+{\text{Li}}_{2}\text{O}.$$

During the following anodic scan, a broad oxidation peak at 0.56 V is in accordance with the dealloying of Li*_x_*Sn [42] and the weak oxidation peak at 1.42 V in the first three cycles can be attributed to the partial delithiation of Li_2_O [43]. The corresponding reaction is as follows:

3.2 $$\text{Sn}+\text{xLi}+{\text{xe}}^{-}⇋{\text{Li}}_{x}\text{Sn}.$$

After the first scan, the reduction/oxidation peaks slightly decreases and the CVs are almost constant, suggesting their good stability. Figure 6*b* exhibits the discharge/charge curves of CNF/SnO_2_/Ni nanocomposite after the first three cycles at 200 mAg^−1^. The CNF/SnO_2_/Ni electrode shows the plateau over a range of 0.5–0.9 V, corresponding to the CV results. The initial discharge/charge capacities of the CNF/SnO_2_/Ni nanocomposite are 1448 and 754 mA hg^−1^. The initial coulombic efficiency is 52%, which is better than the CNF/SnO_2_ sample (electronic supplementary material, figure S6). The initial irreversible capacity loss could be due to the formation of SEI layers originating from electrolyte decomposition [44].

Cycling performance of CNF/SnO_2_/Ni is carried out at a current density of 200 mA g^−1^ from 0.01 to 3.0 V (as shown in figure 6*c*). The CNF/SnO_2_/Ni nanocomposite display a higher capacity (542.8 mA h g^−1^) than those of CNF/SnO_2_ (267.4 mA h g^−1^) (electronic supplementary material, figure S7). CNF/SnO_2_/Ni is able to keep a reversible capacity of around 421.2 mA h g^−1^ with a high coulombic efficiency of 100% after 100 cycles, which implies that the good performance of the CNF/SnO_2_/Ni nanocomposite is attributed to high theoretical capacity and the synergistic effect of CNF/SnO_2_ and Ni, leading to the highly reversible decomposition of Li_2_O [45]. The morphology of CNF/SnO_2_/Ni remains literally unchanged after 100 discharge/charge cycles as shown by the TEM image (electronic supplementary material, figure S8). To obtain insight into the electrochemical kinetics of the CNF/SnO_2_/Ni electrode, the EIS measurement is conducted from 1 Hz to 1 MHz, in which Z′ and Z′′ are the real and imaginary parts of the impedance, respectively. Obviously, one semicircle and a sloping straight line show in the high-and low-frequency range, corresponding to the ohmic resistance and the transfer of lithium ions, respectively (figure 6*d*). The EIS result confirms that the CNF/SnO_2_/Ni sample has low charge-transfer resistances. The CNF/SnO_2_/Ni sample (B) obtained by milling the mixture of CNF/SnO_2_ and Ni powder with a weight ratio 4 : 1 was also synthesized to investigate the effect of Ni content. The results show that CNF/SnO_2_/Ni with a weight ratio 4 : 1 has the similar structural and electrochemical properties with the exception of the different Raman peak intensity and detailed experimental data can be found in the electronic supplementary material. The CNF/SnO_2_/Ni electrode shows good rate capability tests at low discharge/charge current density (as shown in electronic supplementary material, figure S9). However, at high current density, the CNF/SnO_2_/Ni electrode cannot retain the capacity retention of 50% compared to the retention at 0.2 A g^−1^, which can be attributed to the small surficial area and low porosity.

In order to investigate the effects of CNF and Ni nanoparticles, a series of experiments have been performed. These results show that capacity of the SnO_2_, CNF/SnO_2_ and CNF/SnO_2_/Ni is according to the following trend:

$$\text{Sn}{\text{O}}_{2}<\text{CNF/Sn}{\text{O}}_{2}<\text{CNF/Sn}{\text{O}}_{2}\text{/Ni}.$$

Detailed data can be found in in electronic supplementary material, table S1. This result indicates that CNF can effectively enhance the electrical conductivity and the Ni nanoparticles can successfully inhibit Sn coarsening during cycling and consequent enhancement of the stability of the reversible conversion reactions. The other factor is that Ni nanocrystals can serve as an effective catalyst for decomposition of Li_2_O and oxidation of metallic Sn from SnO_2_ according to the following reactions:

3.3 $$\text{Ni}+\text{L}{\text{i}}_{2}\text{O}\to \text{NiO}+2\text{L}{\text{i}}^{+}+2{\text{e}}^{-}$$

and

3.4 $$\text{NiO}+2\text{L}{\text{i}}^{+}+2{\text{e}}^{-}\to \text{Ni}+\text{L}{\text{i}}_{2}\text{O}.$$

The above-mentioned results clearly demonstrate that the CNF/SnO_2_/Ni nanocomposite is a good anode material for LIBs. This might be attributed to the combined merits of following factors: (1) the groove structure of CNF/SnO_2_/Ni nanocomposite, which can minimize structural distortion during the discharge/charge process; (2) the synergistic effect of CNF/SnO_2_ and Ni, which results in highly reversible conversion reactions; (3) good electrical conductivity of graphitized carbon nanofibres; (4) ultra-small size of SnO_2_ nanocrystals shortening the diffusion path of Li^+^.

## 4. Conclusion

A hierarchical CNF/SnO_2_/Ni Nanostructures of graphitized carbon nanofibres, SnO_2_ nanocrystallines and Ni nanocrystallines has been prepared via a facile and low-cost approach by using commercial PAN fibres as the precursor. The nanocomposite shows good lithium storage capacity and cyclability, which can be attributed to the particular groove structure, synergistic effect of CNF/SnO_2_ and Ni, good electrical conductivity of graphitized carbon nanofibres and ultra-small size of SnO_2_ nanocrystals shortening the diffusion path of Li^+^. This work can provide a new approach to the development of conversion-type electrode materials for LIBs and other advanced applications on the basis of PAN.

## Supplementary Material

The supplement information

## Data accessibility

We have conducted our experiment systematically and reported the experimental procedure clearly in the experimental section and provided all necessary data in the main manuscript and supporting information.

## References

[1] Park CM, Kim JH, Kim H, Sohn HJ 2010 Li-alloy based anode materials for Li secondary batteries. Chem. Soc. Rev. 39, 3115–3141. (doi:10.1039/B919877F) 20593097 10.1039/b919877f

[2] Liu N, Wu H, McDowell MT, Yao Y, Wang C, Cui Y 2012 A yolk-shell design for stabilized and scalable Li-ion battery alloy anodes. Nano Lett. 12, 3315–3321. (doi:10.1021/nl3014814) 22551164 10.1021/nl3014814

[3] Choi NS et al. 2012 Challenges facing lithium batteries and electrical double-layer capacitors. Angew. Chem. Int. Ed. 51, 9994–10024. (doi:10.1002/anie.201201429) 10.1002/anie.201201429 22965900

[4] Sun J, Lv CH, Lv F, Chen S, Li DH, Guo Z, Han W, Yang DJ, Guo SJ 2017 Tuning the shell number of multi-shelled metal oxide hollow fibers for optimized lithium ion storage. ACS Nano 11, 6186–6193. (doi:10.1021/acsnano.7b02275) 28505426 10.1021/acsnano.7b02275

[5] Tarascon JM, Armand M 2001 Issues and challenges facing rechargeable lithium batteries. Nature 414, 359–367. (doi:10.1038/35104644) 11713543 10.1038/35104644

[6] Mao XJ, Li JQ, Han C, Yao SS, Zhang J, Zhai HA, Chen LL, Shen XQ, Xiao KS 2017 Electrospinning preparation of oxygen-deficient nano TiO_2-x_/carbon fibre membrane as a self-standing high performance anode for Li-ion batteries. R. Soc. open sci. 4, 170323 (10.1098/rsos.170323) 28791160 10.1098/rsos.170323 PMC5541555

[7] Zhang WM, Hu JS, Guo YG, Zheng SF, Zhong LS, Song WG, Wan LJ 2008 Tin-nanoparticles encapsulated in elastic hollow carbon spheres for high-performance anode material in lithium-Ion batteries. Adv. Mater. 20, 1160–1165. (doi:10.1002/adma.200701364)

[8] Chan CK, Patel RN, O'connell MJ, Korgel BA, Cui Y 2010 Solution-grown silicon nanowires for lithium-ion battery anodes. ACS nano 4, 1443–1450. (doi:10.1021/nn901409q) 20201547 10.1021/nn901409q

[9] Goriparti S, Miele E, De Angelis F, Di Fabrizio E, Zaccaria RP, Capiglia C 2014 Review on recent progress of nanostructured anode materials for Li-ion batteries. J. Power Sources 257, 421–443. (doi:10.1016/j.jpowsour.2013.11.103)

[10] Kim C, Noh M, Choi M, Cho J, Park B 2005 Critical size of a nano SnO_2_ electrode for Li-secondary battery. Chem. Mater. 17, 3297–3301. (doi:10.1021/cm048003o)

[11] Yin XM, Li CC, Zhang M, Hao QY, Liu S, Chen LB, Wang TH 2010 One-step synthesis of hierarchical SnO_2_ hollow nanostructures via self-assembly for high power lithium ion batteries. J. Phys. Chem. C 114, 8084–8088. (doi:10.1021/jp100224x)

[12] Park MS, Wang GX, Kang YM, Wexler D, Dou SX, Liu HK 2007 Preparation and electrochemical properties of SnO_2_ nanowires for application in lithium-ion batteries. Angew. Chem. 119, 764–767. (doi:10.1002/ange.200603309) 10.1002/anie.200603309 17163569

[13] Zhang M, Lei DN, Du ZF, Yin XM, Chen LB, Li QH, Wang YG, Wang TH 2011 Fast synthesis of SnO_2_/graphene composites by reducing graphene oxide with stannous ions. J. Mater. Chem. 21, 1673–1676. (doi:10.1039/C0JM03410J)

[14] Wang XL, Li JZ, Chen Z, Lei LJ, Liao XP, Huang X, Shi B 2016 Hierarchically structured C@SnO_2_@C nanofiber bundles with high stability and effective ambipolar diffusion kinetics for high-performance Li-ion batteries. J. Mater. Chem. A 4, 18 783–18 791. (doi:10.1039/C6TA06622D)

[15] Wang J, Fang F, Yuan T, Yang JH, Chen L, Yao C, Zheng SY, Sun DL 2017 Three-dimensional graphene/single-walled carbon nanotube aerogel anchored with SnO_2_ nanoparticles for high performance lithium storage. ACS Appl. Mater. Interfaces 9, 3544–3553. (doi:10.1021/acsami.6b10807) 28060478 10.1021/acsami.6b10807

[16] Vaughn DD, Hentz OD, Chen SR, Wang DH, Schaak RE 2012 Formation of SnS nanoflowers for lithium ion batteries. Chem. Commun. 48, 5608–5610. (doi:10.1039/C2CC32033A) 10.1039/c2cc32033a 22547153

[17] Kim TJ, Kim C, Son D, Choi M, Park B 2007 Novel SnS_2_-nanosheet anodes for lithium-ion batteries. J. Power Sources 167, 529–535. (doi:10.1016/j.jpowsour.2007.02.040)

[18] Lu J, Nan CY, Li LH, Peng Q, Li YD 2013 Flexible SnS nanobelts: facile synthesis, formation mechanism and application in Li-ion batteries. Nano Res. 6, 55–64. (doi:10.1007/s12274-012-0281-7)

[19] Li S, Zheng JX, Zuo SY, Wu ZG, Yan PX, Pan F 2015 2D hybrid anode based on SnS nanosheet bonded with graphene to enhance electrochemical performance for lithium-ion batteries. Rsc Adv. 5, 46 941–46 946. (doi:10.1039/C5RA07292A)

[20] Du YP et al. 2013 A facile, relative green, and inexpensive synthetic approach toward large-scale production of SnS_2_ nanoplates for high-performance lithium-ion batteries. Nanoscale 5, 1456–1459. (doi:10.1039/C2NR33458E) 23306599 10.1039/c2nr33458e

[21] Tian F, Wang XB, Chen ZY, Guo YM, Liang HJ, Lu ZS, Wang D, Lou XD, Yang L 2016 A facile post-process method to enhance crystallinity and electrochemical properties of SnO_2_/rGO composites with three-dimensional hierarchically porous structure. RSC Adv. 6, 106 275–106 284. (doi:10.1039/C6RA23236A)

[22] Wang ZY, Luan DY, Boey FYC, Lou XW 2011 Fast formation of SnO_2_ nanoboxes with enhanced lithium storage capability. J. Am. Chem. Soc. 133, 4738–4741. (doi:10.1021/ja2004329) 21401090 10.1021/ja2004329

[23] Wang C, Zhou Y, Ge MY, Xu XB, Zhang ZL, Jiang JZ 2010 Large-scale synthesis of SnO_2_ nanosheets with high lithium storage capacity. J. Am. Chem. Soc. 132, 46–47. (doi:10.1021/ja909321d) 20000321 10.1021/ja909321d

[24] Ding SJ, Luan DY, Boey FYC, Chen JS, Lou XWD 2011 SnO_2_ nanosheets grown on graphene sheets with enhanced lithium storage properties. Chem. Commun. 47, 7155–7157. (doi:10.1039/C1CC11968K) 10.1039/c1cc11968k 21607244

[25] Lou XW, Wang Y, Yuan C, Lee JY, Archer LA 2006 Template-free synthesis of SnO_2_ hollow nanostructures with high lithium storage capacity. Adv. Mater. 18, 2325–2329. (doi:10.1002/adma.200600733)

[26] Hassan FM, Hu QQ, Fu J, Batmaz R, Li JD, Yu AP, Xiao XC, Chen ZW 2017 Hot-chemistry structural phase transformation in single crystal chalcogenides for long-life lithium-ion batteries. ACS Appl. Mater. Interfaces 9, 20 603–20 612. (doi:10.1021/acsami.7b04483) 10.1021/acsami.7b04483 28557416

[27] Zhou XS, Wan LJ, Guo YG 2013 Binding SnO_2_ nanocrystals in nitrogen-doped graphene sheets as anode materials for lithium-ion batteries. Adv. Mater. 25, 2152–2157. (doi:10.1002/adma.201300071) 23427163 10.1002/adma.201300071

[28] Jiang Y, Feng YZ, Xi BJ, Kai SS, Mi K, Feng JK, Zhang JH, Xiong SL 2016 Ultrasmall SnS_2_ nanoparticles anchored on well-distributed nitrogen-doped graphene sheets for Li-ion and Na-ion batteries. J. Mater. Chem. A 4, 10 719–10 726. (doi:10.1039/C6TA03580A)

[29] Jiang Y, Wei M, Feng JK, Ma YC, Xiong SL 2016 Enhancing the cycling stability of Na-ion batteries by bonding SnS_2_ ultrafine nanocrystals on amino-functionalized graphene hybrid nanosheets. Energy Environ. Sci. 9, 1430–1438. (doi:10.1039/C5EE03262H)

[30] Luo B, Fang Y, Wang B, Zhou JS, Song HH, Zhi LJ 2012 Two dimensional graphene–SnS_2_ hybrids with superior rate capability for lithium ion storage. Energy Environ. Sci. 5, 5226–5230. (doi:10.1039/C1EE02800F)

[31] Mei L, Xu C, Yang T, Ma JM, Chen LB, Li QH, Wang TH 2013 Superior electrochemical performance of ultrasmall SnS_2_ nanocrystals decorated on flexible RGO in lithium-ion batteries. J. Mater. Chem. A 1, 8658–8664. (doi:10.1039/C3TA11269A)

[32] Zhou D, Song WL, Fan LZ 2015 Hollow core–shell SnO_2_/C fibers as highly stable anodes for lithium-ion batteries. ACS appl. Mater. Iinterfaces 7, 21 472–21 478. (doi:10.1021/acsami.5b06512) 10.1021/acsami.5b06512 26348195

[33] Kim D, Lee D, Kim J, Moon J 2012 Electrospun Ni-added SnO_2_–carbon nanofiber composite anode for high-performance lithium-ion batteries. ACS appl. Mater. Interfaces 4, 5408–5415. (doi:10.1021/am301328u) 22999049 10.1021/am301328u

[34] Guo H, Zhao H, Jia X 2007 Spherical Sn–Ni–C alloy anode material with submicro/micro complex particle structure for lithium secondary batteries. Electrochem. Commun. 9, 2207–2211. (doi:10.1016/j.elecom.2007.06.021)

[35] Hu RH, Ouyang YP, Liang T, Wang H, Liu J, Chen J, Yang CH, Yang LC, Zhu M 2017 Stabilizing the nanostructure of SnO_2_ anodes by transition metals: a route to achieve high initial coulombic efficiency and stable capacities for lithium storage. Adv. Mater. 29, 1605006 (doi:10.1002/adma.201605006) 10.1002/adma.201605006 28185334

[36] Chappé JM et al. 2011 Structure and chemical bonds in reactively sputtered black Ti–C–N–O thin films. Thin Solid Films 520, 144–151. (doi:10.1016/j.tsf.2011.06.108)

[37] Wang Y, Zou YC, Chen J, Li GD, Xu Y 2013 A flexible and monolithic nanocomposite aerogel of carbon nanofibers and crystalline titania: fabrication and applications. RSC Adv. 3, 24 163–24 168. (doi:10.1039/C3RA44820G)

[38] Zhang F, Cao HQ, Yue DM, Zhang JX, Qu MZ 2012 Enhanced anode performances of polyaniline–TiO_2_–reduced graphene oxide nanocomposites for lithium ion batteries. Inorg. Chem. 51, 9544–9551. (doi:10.1021/ic301378j) 22906577 10.1021/ic301378j

[39] Zhu J, Yang J, Bian ZF, Ren J, Liu YM, Cao Y, Li HX, He HY, Fan KN 2007 Nanocrystalline anatase TiO_2_ photocatalysts prepared via a facile low temperature nonhydrolytic sol–gel reaction of TiCl_4_ and benzyl alcohol. Appl. Catal. B Environ. 76, 82–91. (doi:10.1016/j.apcatb.2007.05.017)

[40] Jing LQ, Sun XJ, Cai WM, Xu ZL, Du YG, Fu HG 2003 The preparation and characterization of nanoparticle TiO_2_/Ti films and their photocatalytic activity. J. Phys. Chem. Solids 64, 615–623. (doi:10.1016/S0022-3697(02)00362-1)

[41] Yan Y, Chen T, Zou Y, Wang Y 2016 Biotemplated synthesis of Au loaded Sn-doped TiO_2_ hierarchical nanorods using nanocrystalline cellulose and their applications in photocatalysis. J. Mater. Res. 31, 1383–1392. (doi:10.1557/jmr.2016.128)

[42] Tao HC, Yang XL, Zhang LL, Ni SB 2014 One-step in situ synthesis of SnS/graphene nanocomposite with enhanced electrochemical performance for lithium ion batteries. J. Electroanal. Chem. 728, 134–139. (doi:10.1016/j.jelechem.2014.07.004)

[43] Shan JQ, Liu YX, Liu P, Huang YS, Su YZ, Wu DQ, Feng XL 2015 Nitrogen-doped carbon-encapsulated SnO_2_-SnS/graphene sheets with improved anodic performance in lithium ion batteries. J. Mater. Chem. A 3, 24 148–24 154. (doi:10.1039/C5TA06617D)

[44] Liang J, Yu XY, Zhou H, Wu HB, Ding SJ, Lou XWD 2014 Bowl-like SnO_2_@carbon hollow particles as an advanced anode material for lithium-ion batteries. Angew. Chem. Int. Ed. 53, 12 803–12 807. (doi:10.1002/anie.201407917) 10.1002/anie.201407917 25251871

[45] Wang CM et al. 2011 *In situ* transmission electron microscopy observation of microstructure and phase evolution in a SnO_2_ nanowire during lithium intercalation. Nano Lett. 11, 1874–1880. (doi:10.1021/nl200272n) 21476583 10.1021/nl200272n

