# Supplementary material for: Facile synthesis of hierarchical CNF/SnO2/Ni nanostructures via self-assembly process as anode materials for lithium ion batteries
Source: R Soc Open Sci. 2018 Jun 20;5(6):171522. doi: 10.1098/rsos.171522 (PMC6030287; doi:10.1098/rsos.171522)
Supplement: The supplement information [file rsos171522supp1.docx]

Facile Synthesis of Hierarchical CNF/SnO_2_/Ni Nanostructures via Self-Assembly Process as Anode Materials for Lithium Ion Batteries

Haitong Tang^1,2^, Xinru Yu^2^, Shi Jin ^2,3^, Fanling Meng^1^, Yan Yan^2^,* Zhongmin Gao^2^,*

*^1^Key Laboratory of Automobile Materials, Ministry of Education, College of Materials Science and Engineering, Jilin University, 2699 Qianjin Street, Changchun, 130012, People’s Republic of China,*

*^2^State Key Laboratory of Inorganic Synthesis and Preparative Chemistry, College of Chemistry, Jilin University,* *2699 Qianjin Street, Changchun, 130012, People’s Republic of China,*

*^3^Jilin Jianzhu University, 5088 Xincheng Street, Changchun, 130018, People’s Republic of China,*

**Supplementary Information**

**Contents:**

Fig. S1 (a) FESEM image of the grinding CNF/SnO_2_ nanoparticles

(b) FESEM image of the CNF

Fig. S2 (a) The EDS spectrum of milling the mixture of CNF/SnO_2_ and Ni powder with weight ratio 10:1.

(b) The EDS spectrum of milling the mixture of CNF/SnO_2_ and Ni powder with weight ratio 4:1

Fig. S3 TGA and Raman spectrum of (a) CNF/SnO_2_/Ni hierarchical nanocomposite.

Fig. S4 XPS spectra of the CNF/SnO_2_ nanocomposite. (a) Survey spectrum, (b) C 1s, (c) O 1s, (d) Sn 3d.

Fig. S5 (a) Nitrogen adsorption-desorption isotherms of the hierarchical porous structure of CNF/SnO2/Ni hierarchical nanocomposite, (b) the pore size distribution of CNF/SnO2/Ni hierarchical nanocomposite.

Fig. S6 Charge-discharge cycle of CNF/SnO_2_ at a rate of 200 mAg^-1^ between 0.01V to 3.0 V.

Fig. S7 Cycling performance cycled at a rate of 200 mAg^-1^.

Fig. S8 TEM image of the CNF/SnO_2_/Ni nanocomposite after 100 discharge/charge cycles.

Fig. S9 The capacity over cycling at different rates (0.1 Ag^-1^ ~ 5 Ag^-1^) of milling the mixture of CNF/SnO_2_ and Ni powder with weight ratio 10:1 and 4:1.

Fig. S10 Charge-discharge cycle of SnO_2_ at a rate of 200 mAg-1 between 0.01V to 3.0 V.

Fig. S11 The CNF/SnO_2_/Ni sample (B) of milling the mixture of CNF/SnO_2_ and Ni powder with weight ratio 4:1 was also synthesized to investigate the effect of nickel content. FESEM image of (B) (Fig. S11a), XPS spectra of the (B) (Fig. S11b), charge-discharge cycle of (B) at a rate of 200 mAg^-1^ between 0.01V to 3.0 V. (Fig. S11c ), Raman spectrum of (B) (Fig. S11d).

Tab. S1 The capacity of CNF/SnO2/Ni, CNF/SnO_2_ and SnO_2_.


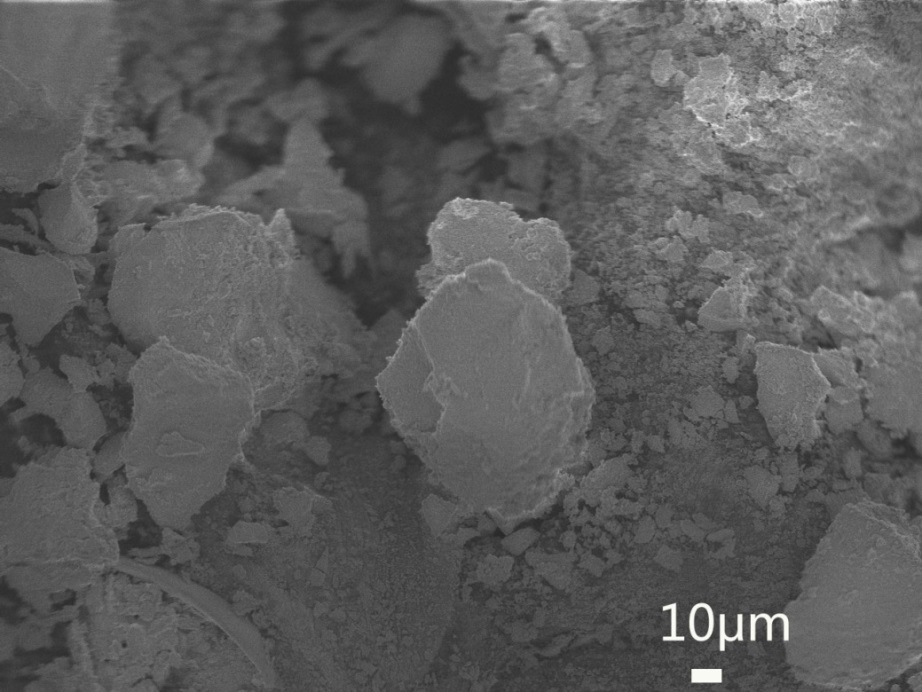


Fig. S1a


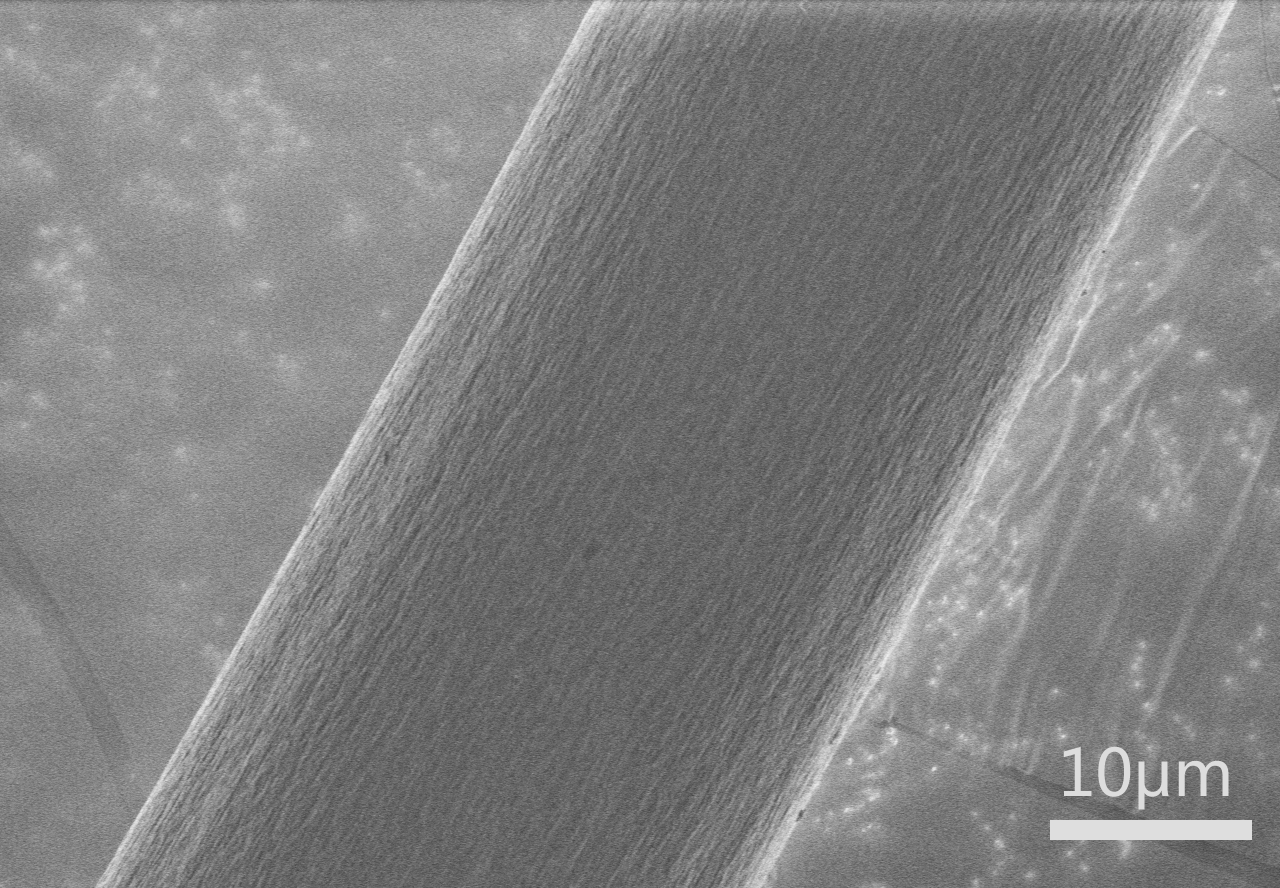


Fig. S1b


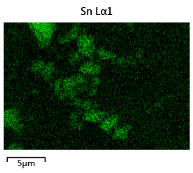

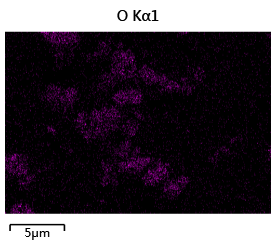


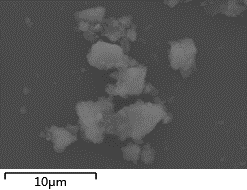


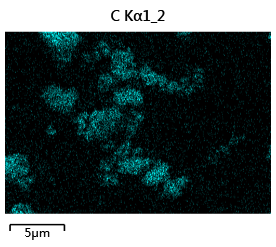

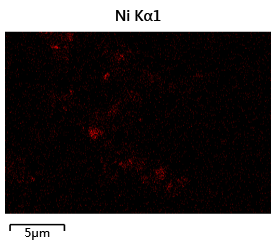


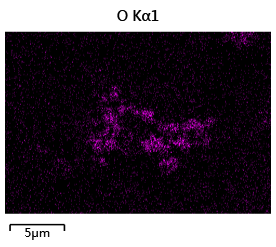

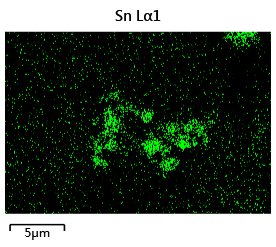
Fig. S2a


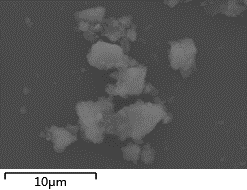


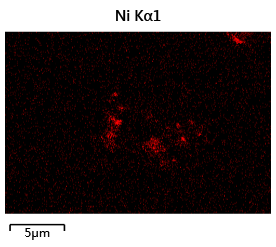

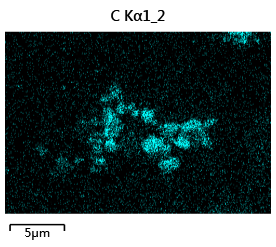


Fig. S2b


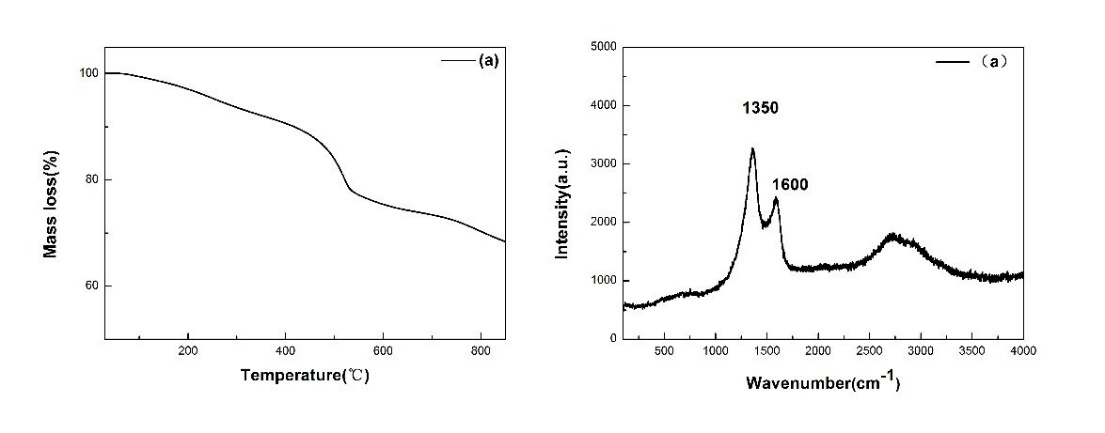


Fig. S3


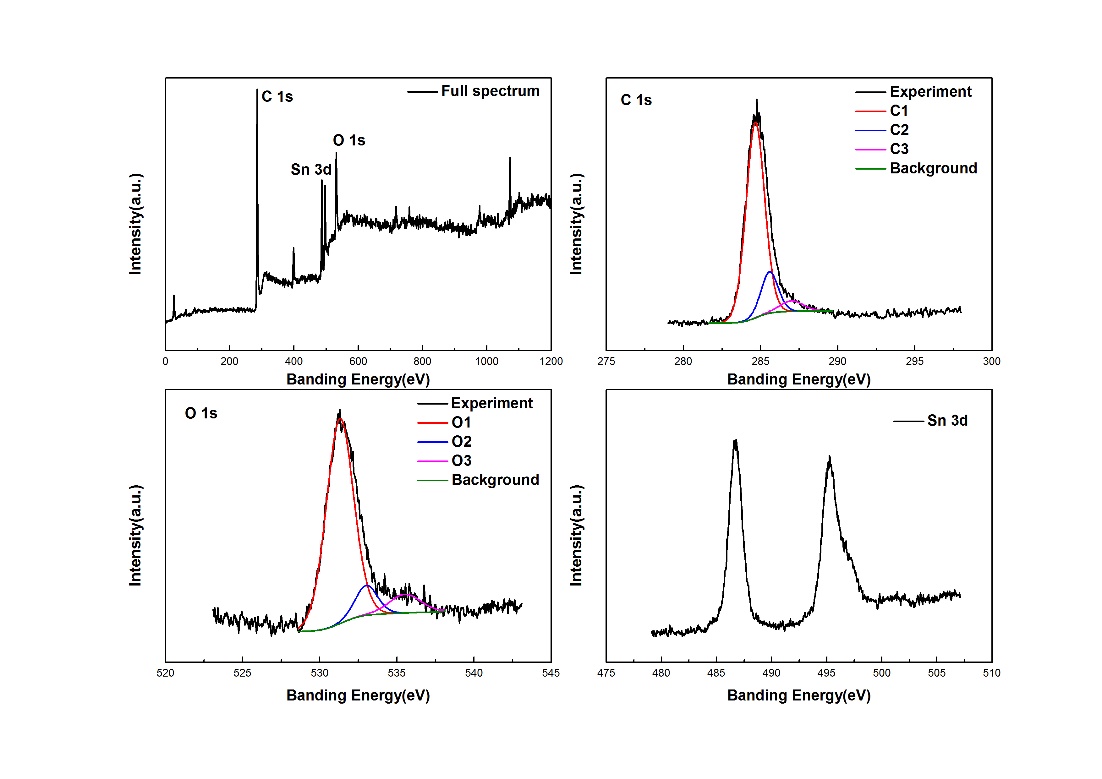


Fig. S4


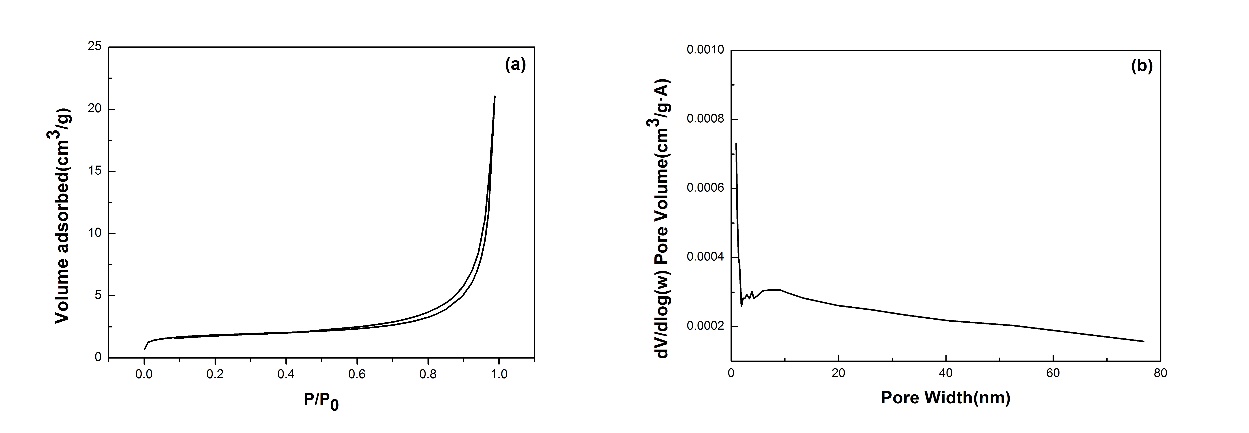


Fig. S5


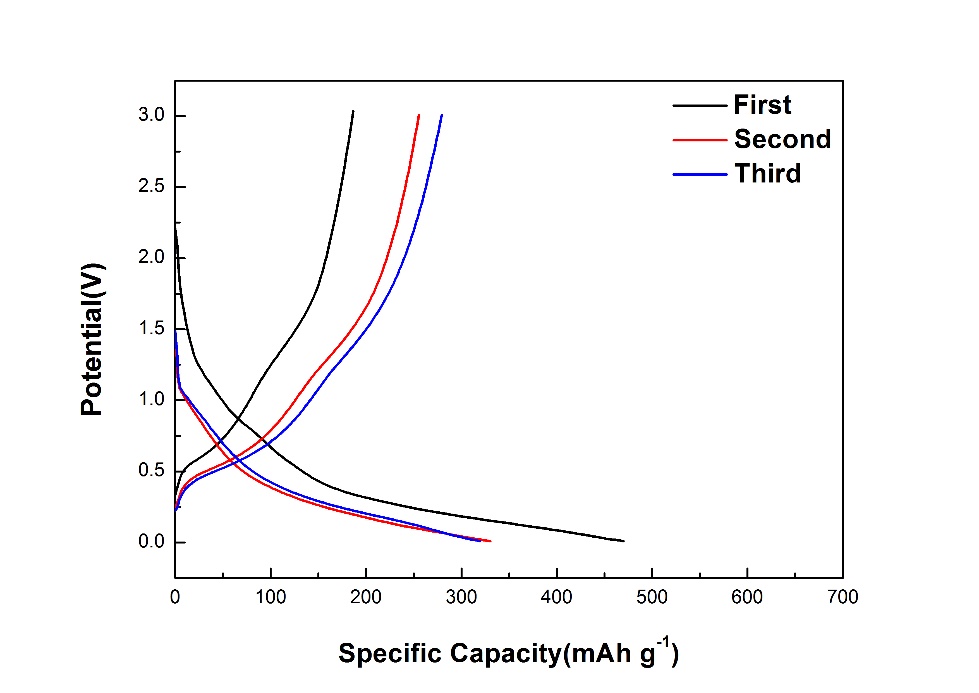


Fig. S6


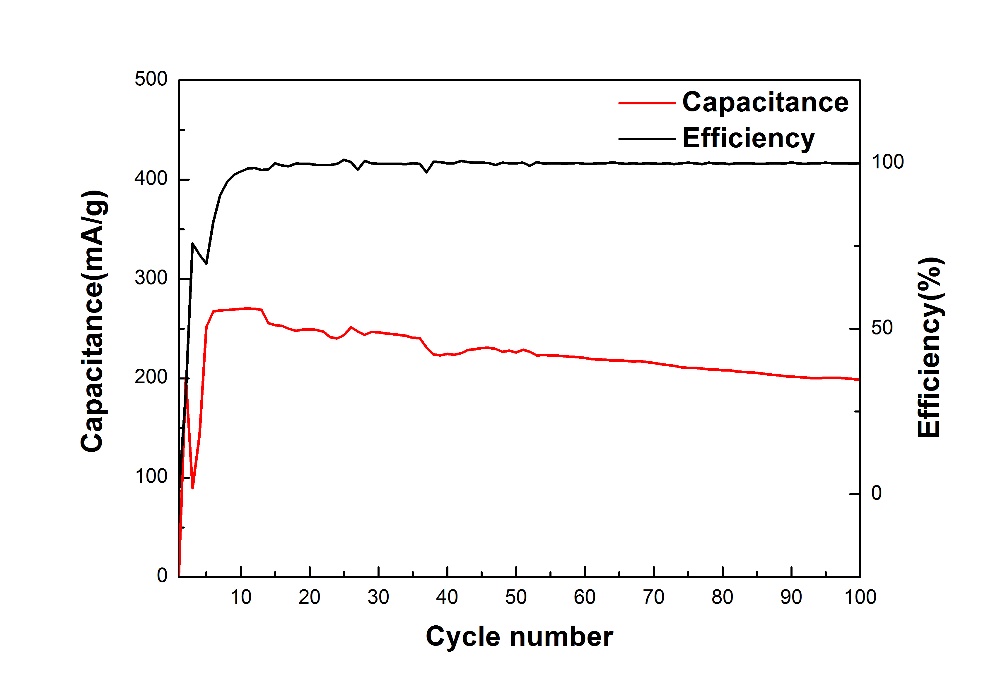


Fig. S7


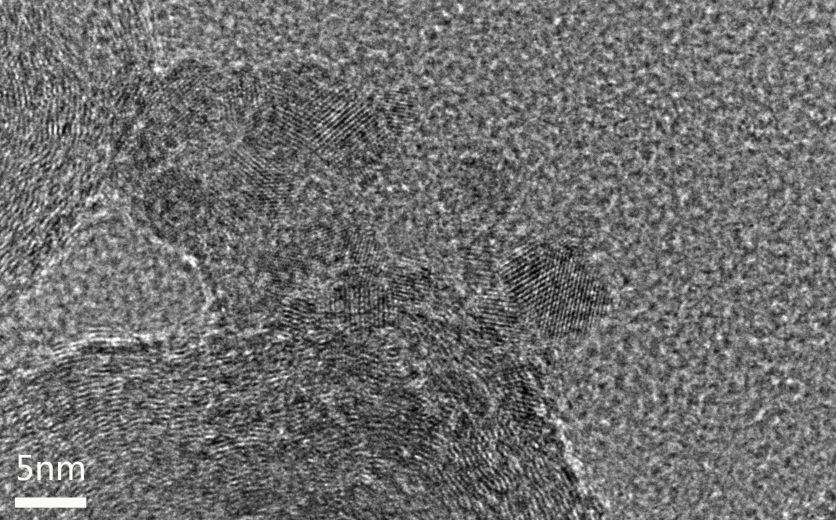


Fig. S8


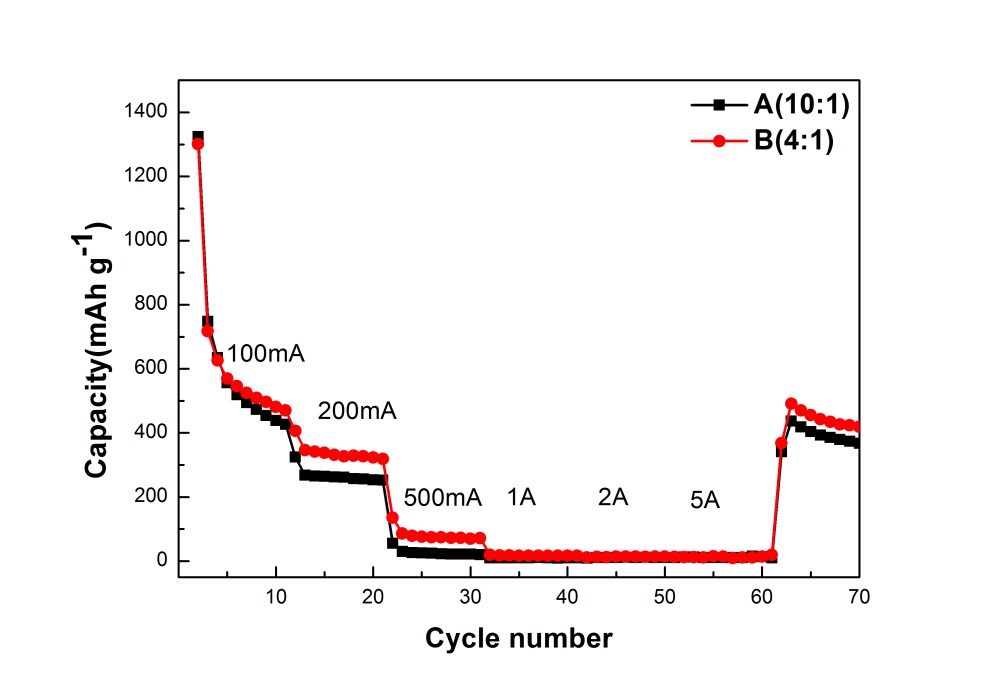


Fig. S9


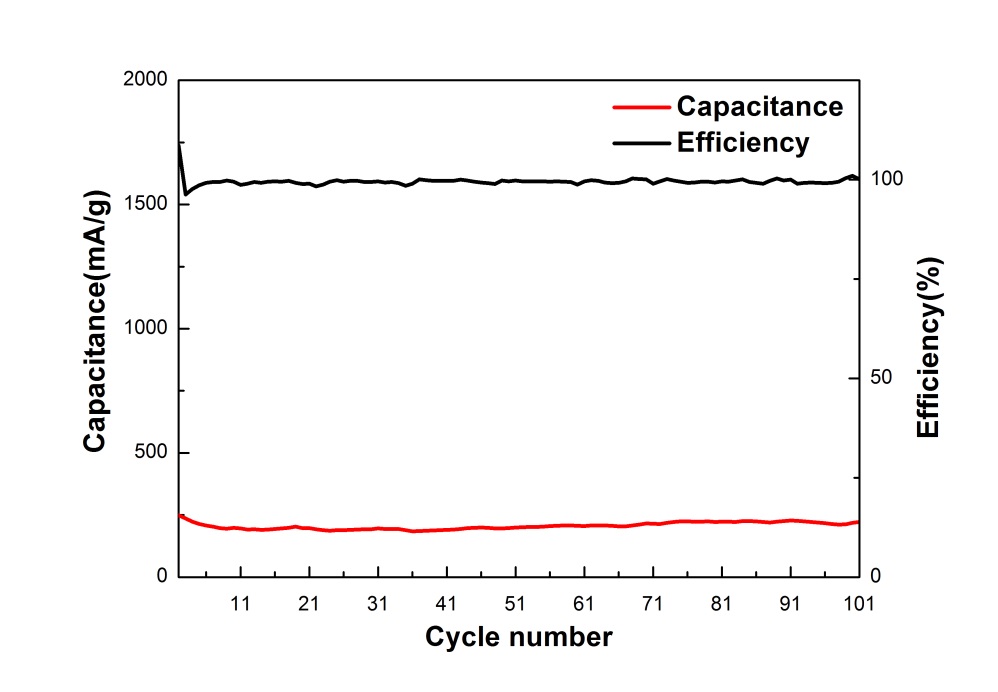


Fig. S10


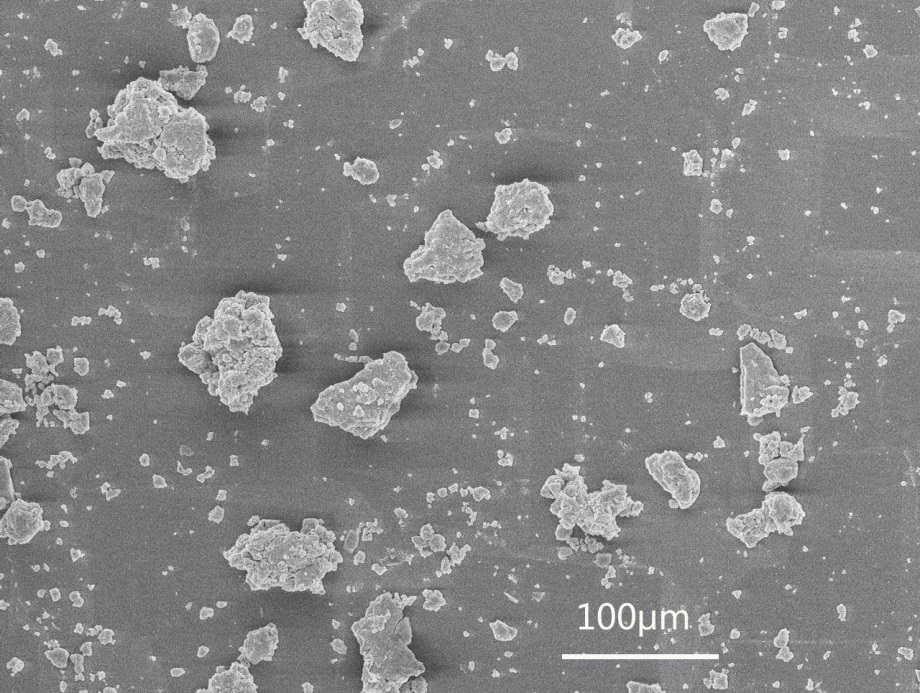


Fig. S11a


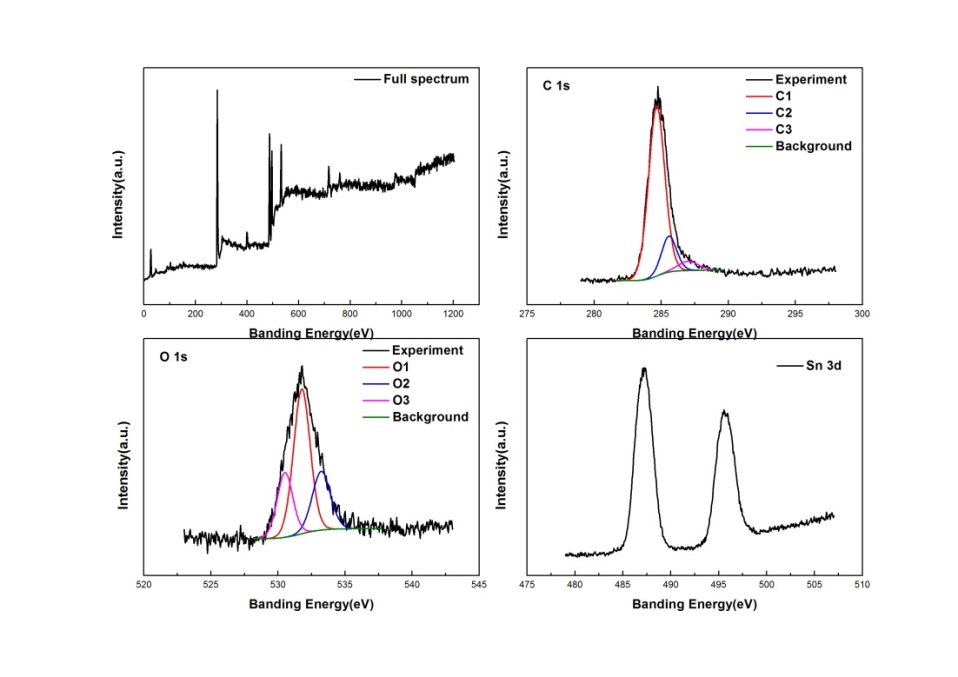


Fig. S11b


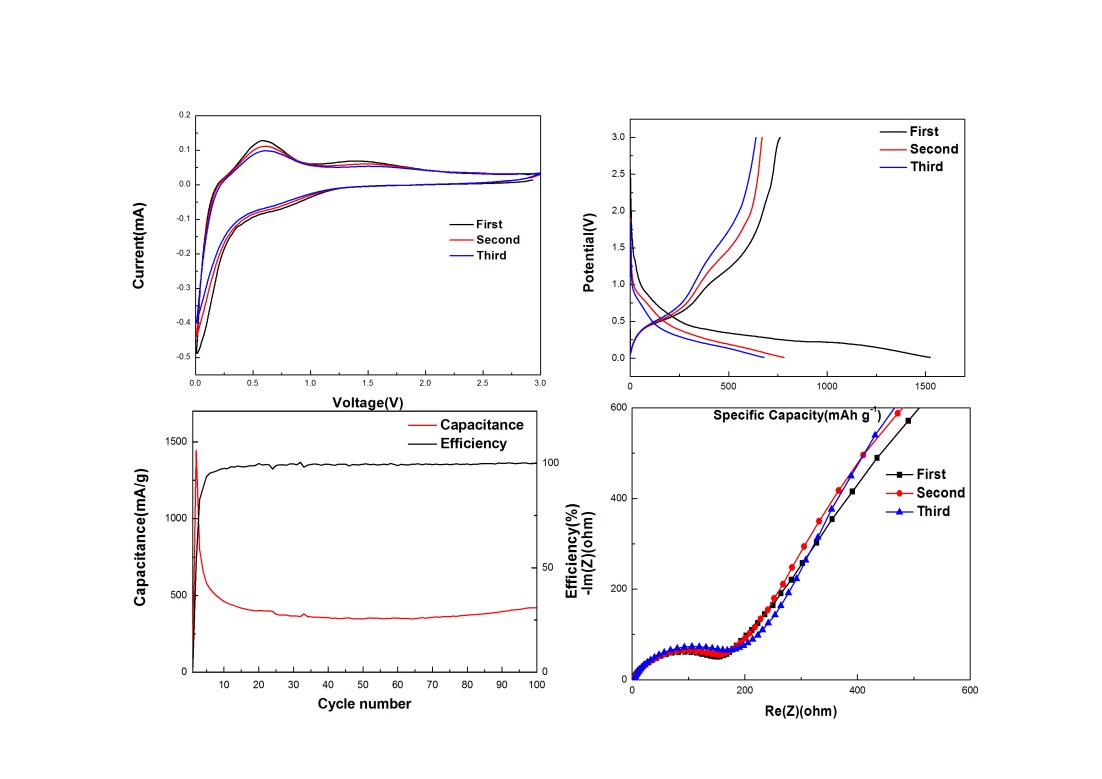


Fig. S11c


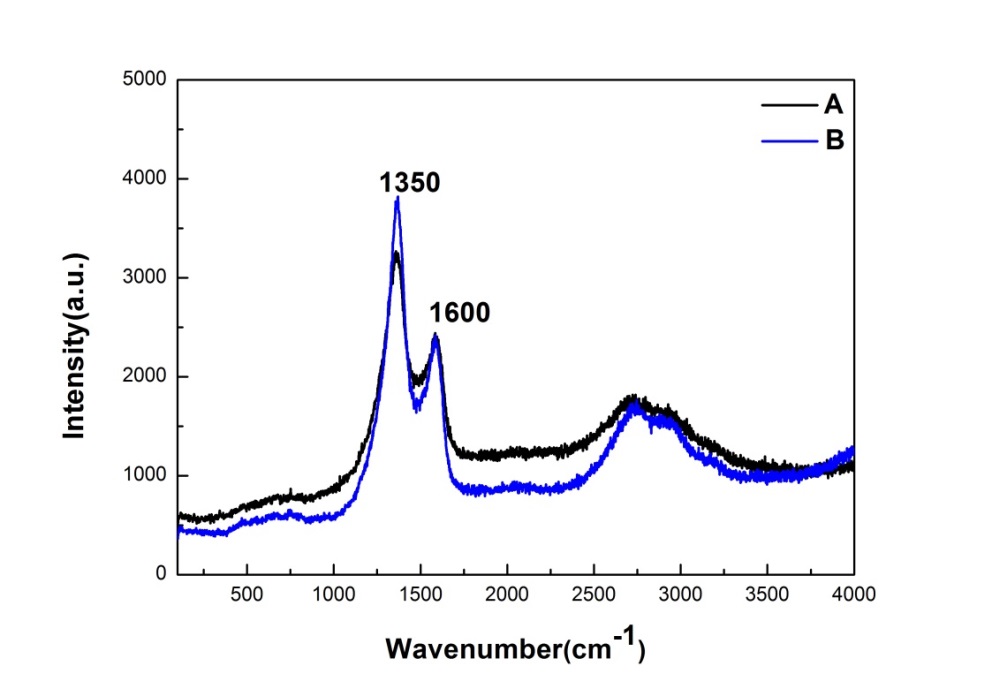


Fig. S11d

Tab. S1 The capacity of CNF/SnO_2_/Ni, CNF/SnO_2_ and SnO_2_.

| Sample | Capacity(mA h g^-1^) |
| --- | --- |
| CNF/SnO_2_/Ni | 542.8 |
| CNF/SnO_2_ | 267.4 |
| SnO_2_ | 248.7 |
